# DNA-binding sequence specificity of DUX4

**DOI:** 10.1186/s13395-016-0080-z

**Published:** 2016-01-28

**Authors:** Yu Zhang, John K. Lee, Erik A. Toso, Joslynn S. Lee, Si Ho Choi, Matthew Slattery, Hideki Aihara, Michael Kyba

**Affiliations:** 1 Lillehei Heart Institute, University of Minnesota, Minneapolis, MN 55455 USA; 2 Department of Pediatrics, University of Minnesota, Minneapolis, MN 55455 USA; 3 Department of Biochemistry, Molecular Biology and Biophysics, University of Minnesota, Minneapolis, MN 55455 USA; 4 Research Center, Dongnam Institute of Radiological & Medical Sciences (DIRAMS), Busan, South Korea; 5 Department of Biomedical Sciences, University of Minnesota Medical School, Duluth, MN 55812 USA

**Keywords:** DUX4, Facioscapulohumeral muscular dystrophy, FSHD, SELEX

## Abstract

**Background:**

Misexpression of the double homeodomain transcription factor DUX4 results in facioscapulohumeral muscular dystrophy (FSHD). A DNA-binding consensus with two tandem TAAT motifs based on chromatin IP peaks has been discovered; however, the consensus has multiple variations (flavors) of unknown relative activity. In addition, not all peaks have this consensus, and the Pitx1 promoter, the first DUX4 target sequence mooted, has a different TAAT-rich sequence. Furthermore, it is not known whether and to what extent deviations from the consensus affect DNA-binding affinity and transcriptional activation potential.

**Results:**

Here, we take both unbiased and consensus sequence-driven approaches to determine the DNA-binding specificity of DUX4 and its tolerance to mismatches at each site within its consensus sequence. We discover that the best binding and the greatest transcriptional activation are observed when the two TAAT motifs are separated by a C residue. The second TAAT motif in the consensus sequence is actually (T/C)AAT. We find that a T is preferred here. DUX4 has no transcriptional activity on “half-sites”, i.e., those bearing only a single TAAT motif. We further find that DUX4 does not bind to the TAATTA motif in the *Pitx1* promoter, that *Pitx1* sequences have no competitive band shift activity, and that the *Pitx1* sequence is transcriptionally inactive, calling into question *PITX1* as a DUX4 target gene. Finally, by multimerizing binding sites, we find that DUX4 transcriptional activation demonstrates tremendous synergy and that at low DNA concentrations, at least two motifs are necessary to detect a transcriptional response.

**Conclusions:**

These studies illuminate the DNA-binding sequence preferences of DUX4.

**Electronic supplementary material:**

The online version of this article (doi:10.1186/s13395-016-0080-z) contains supplementary material, which is available to authorized users.

## Background

Facioscapulohumeral muscular dystrophy (FSHD) is arguably the most prevalent genetic disease of muscle [1, 2]. It is caused by altered regulation of the subtelomeric chr4q macrosatellite repeat, D4Z4 [3–6]. This 3.3-kb macrosatellite sequence is typically present in ~30 tandem copies [7], while most cases of FSHD involve array contractions bringing the number of tandem repeats down to 10 or fewer [8, 9]. When this occurs, the array, which is normally silent through a poorly understood repeat-induced silencing mechanism, becomes transcriptionally active [10, 11]. Alternatively, the array can become transcriptionally active through second site mutations in genes required for repeat-induced silencing, for example, SMCHD1 [12–14]. When this happens in the context of an allele that provides a downstream polyA signal [15], a muscle pathology ensues.

The D4Z4 repeat contains an open reading frame encoding DUX4, a double-homeodomain transcription factor [16]. The DUX4 protein is quite difficult to detect in FSHD clinical specimens [17, 18], but its presence can be read out indirectly in both proliferating and differentiating myoblasts, with slightly greater expression in the latter [19, 20]. When induced at low levels of expression in myogenic progenitor cells, DUX4 interferes with MyoD expression and impairs myogenic differentiation [21, 22]. High levels of DUX4 expression promote cell death [22, 23]. As the homeodomains of DUX4 fall within the paired homeodomain class, and indeed are very close in sequence to those of the skeletal muscle stem cell regulators Pax3 and Pax7 [24, 25], a model suggesting that skeletal myogenic phenotypes in FSHD may be due in part to competition with Pax3/7 for targets was proposed [22]. Supporting this possibility, overexpression of either Pax3 or Pax7, but not the more distantly related homeodomain protein HoxB4, significantly reduced the cytotoxicity of DUX4 [22]. The sequence binding preferences of both DUX4 and Pax3/7 have recently been described through interrogation of sequences falling under ChIP-seq peaks [11, 26]. Although both sequences contain TAAT core motifs, they differ in that the DUX4 consensus site contains two TAAT motifs in tandem, while the Pax3/7 site contains the motifs in a head-to-head orientation (i.e., TAAT followed by ATTA). Besides binding sequences identified through ChIP-seq, DUX4 binding to a sequence in the promoter of *Pitx1* had been demonstrated by band shift assays [10, 27]. The *Pitx1* sequence does not contain a tandem TAAT motif, but rather has two overlapping head-to-head motifs: TAATTA, and this motif is also present in the human *PITX1* gene. Thus, from work to date, it is not entirely clear what sequences DUX4 can bind to. Specific tests comparing DUX4 activity on different flavors of target sequences have never been done.

Because of the central role that DUX4 plays in FSHD, an understanding of the DNA-binding activity of DUX4 is essential to a mechanistic understanding of the disease. We have taken unbiased and candidate-selected approaches to compare the DNA-binding and transcriptional enhancing activity of DUX4 on various known sequences as well as randomly generated variants. Using the DNA element of greatest potency, we also investigate the copy number dependency of transcriptional activation by DUX4.

## Methods

### Reporter constructs

The luciferase reporter construct pGL4-12X-DUX4 containing 12× DUX4 binding motifs (CT flavor: TAATCTAATCA) was synthesized by GENEWIZ (New Jersey) and subcloned into XhoI/HindIII linearized the pGL4-Amp luciferase plasmid (Promega) using T4 ligation. To generate the 6× reporter, pGL4-6X-DUX4 6 motifs were removed from this construct using KpnI digestion, followed by T4 ligation. To generate the 24× construct, pGL4-24X-DUX4, we ligated an XhoI/SalI fragment from pGL4-12X-DUX4 into XhoI linearized pGL4-12X-DUX4 plasmid and the correct orientation selected. All other luciferase plasmids were constructed by T4 ligation of XhoI/HindIII linearized pGL4-Amp(R) luciferase plasmid with corresponding PCR-amplified fragments using In-Fusion HD cloning (Clontech). PCR fragments and primer information are listed in Additional file 1: Table S1.

### Generation of DUX4-inducible 293T cells

FUIGW-rtTA was constructed by inserting rtTA2(s)-m2 (amplified by PCR) into BamH1/EcoR1 FUIGW (Lyu et al. 2008). pSam2-iDUX4-Flag-UBC-puro, the doxycycline-inducible DUX4 lentivector, was generated in the following way: The polyA signal from SV40 was amplified from p2lox (Iacovino et al. 2011) and inserted into pSAM2 (Zhang et al. 2011) at the Not1 site. The Ubiquitin C promoter and EGFP from FUGW (Lois et al. 2002) was then inserted into Pac1/BsrG1-digested plasmid, replacing the sgTRE promoter. The puromycin resistance gene (PAC) was PCR amplified and used to replace GFP by in-fusion cloning (Clontech). DUX4 with a c-terminal Flag peptide was PCR amplified and inserted into EcoR1/Not1 digested plasmid to generate pSam2-iDUX4-Flag-Ubc-Puro.

### Transfection and luciferase assays

Prior to transient transfection, DUX4-inducible 293T cells were plated in 96-well dishes until cells reached 60 % confluency. Each well of cells was transfected with 95 ng of pGL4 firefly luciferase reporter plasmid together with 5 ng of Renilla luciferase control plasmid using TransIT-LT1 transfection reagent (Mirus Bio LLC). Doxycycline (500 ng/ml) was added into each well after 24-h post-transfection to induce DUX4 expression, and cells were lysed 48 h post-transfection for luciferase assays using the Dual-Glo Luciferase Assay System (Promega). For luciferase assays, 75 μl of Dual-Glo luciferase assay reagent was added to each well and incubated at room temperature for 15 min before measuring the firefly luminescence. After measuring, 75 μl of Dual-Glo Stop & Glo reagent was added into each well and incubated at room temperature for 15 min to quench the firefly luciferase activity, after which Renilla luminescence activity was measured. Luminescence readouts were measured using Cytation 3 plate reader (Bio-Tek) under luminescence fiber mode with the Gain-value fixed at 135. Firefly luminescence was first normalized to Renilla luminescence and scaled to fold of induction to the control well (no dox addition). Each reporter analysis was done in triplicate and repeated twice.

### Bacterial expression of the DUX4 N-terminus

The N-terminus of DUX4, containing the two homeodomains was expressed in bacteria using the plasmid pET28 with a tobacco etch virus nuclear inclusion A endopeptidase protease (TEV) site engineered between the His6 tag and protein. We altered the codon usage, PCR amplified from a synthetic construct (Genscript) using primers:

Dux4_TEV19G_NdeI:

CGGAATTCCATATGgaaaacctgtacttccagggtAGACGTCGCAGGTTAGTTTGGACAC and

Dux4_152Qstop_BamHI:

TCGCGGATCCTTACTGACCAGGGTGACGAGCACGTCTGTTTTG,

and subcloned into the vector.

The DUX4-HD protein was produced in BL21(DE3) and purified by Ni-NTA IMAC affinity purification. The recombinant DUX4 protein (containing the N-terminal His6 tag) was cleaved off with TEV protease, and the final protein was purified by size exclusion chromatography (Superdex 200). For later experiments, we used a His-tagged SUMO-fusion construct (pE-SUMO, LifeSensors) cloned with following primers:

DUX4_15Ser_BsmBI:

CCCCCTGCTAATCCGTCTCAAGGTtctAGAGGTAGAGGTAGACGTCGCAGGTTAG

DUX4_155R_BsmBI/XbaI:

CCCCTGCTAATCCGTCTCTCTAGATTATCTACCACCCTGACCAGGGTGACGAGC

### Band shift assays

Band shift experiments were performed with double stranded oligos of the following sequences:

DUX4(TT) GGCAGTCTAATTTAATCAAGTCGGC

DUX4(CT) GGCAGTCTAATCTAATCAAGTCGGC

DUX4(TC) GGCAGTCTAATTCAATCAAGTCGGC

DUX4(CC) GGCAGTCTAATCCAATCAAGTCGGC

DUX4-del GGCAGTCTAATTAATCAAGTCGGC

DUX4-ins GGCAGTCTAATCTTAATCAAGTCGGC

MALR GGCAGTCTAATTGAATCAAGTCGGC

Pitx1-25 GGCAGTCTTCTAATTAGTAGTCGGC

Pitx1-30 CGGATGCTGTCTTCTAATTAGTTTGGACCC

SELEX2 GGCAGTCTAATTCAATCCAGTCGGC

SELEX3 GGCAGTCTAATTAGCTTTAGTCGGC

SELEX4 GGCAGTCTAATGTTTTATAGTCGGC

#### Noncompetitive band shifts

In a final volume of 30 μL, 10 μL of 100 μM probe was mixed with 20 μL of 250 μM DUX4 HD protein (in 500 mM NaCl, 20 mM Tris-Cl, pH 7.4). Samples were incubated on ice for 1 h, then run immediately on a 3 % agarose gel containing 0.5 μg/ml EtBr.

#### Competitive band shifts

Ten microliters of 100 μM probe was added to 16 μL of milliQ H_2_O; 2 μL of DUX4 HD protein (125 μM in 50 % glycerol, 250 mM NaCl, 10 mM Tris-Cl, pH 7.4) was then added, followed by 2 μL of 100 μM FAM-labelled CT probe (final volume of 30 μL). Samples were incubated on ice for 1 h, then run immediately on a 3 % agarose gel, with no EtBr.

### SELEX-seq: DNA pulldown assays

We synthesized the following partially randomized single stranded oligonucleotides:

Synthetic Bait-1 target: TCGTCGGCAGCGTCAGATGTGTATAAGAGACAGNNNNNNNNTAATNNNNNNNNCTGTCTCTTATACACATCTCCGAGCCCACGAGAC (underlined sequences represent Nextera adapter sequences).

Synthetic Bait-2 target: TCGTCGGCAGCGTCAGATGTGTATAAGAGACAGNNNT’A’A’T’T’T’A’A’T’C’A’NNNCTGTCTCTTATACACATCTCCGAGCCCACGAGAC

A’ refers to a mixture of 91 % dATP, and 3 % each of the other deoxynucleotides and so on for T’, C’, and G’.

We generated double-stranded oligonucleotide from these using 15 cycles of amplification with the following primers: BaitF: TCGTCGGCAGCGTC, BaitR: GTCTCGTGGGCTCGG.

Prior to DNA pulldown, 250 ng of His-tagged DUX4 DNA-binding domain was incubated with 50 μl of Ni-NTA resin (Thermo Scientific) in 500 μl of NT2 buffer (20 mM Tris-HCl (pH 7.5), 100 mM NaCl, 0.05 % Nonidet P-40) at 4 °C for 30 min, followed by three washes using 500 μl of NT2 buffer. Then, 3 μg of bait DNA was incubated with resin-protein complex in 250 μl of binding buffer (20 mM Tris-HCl (pH 7.5), 100 mM NaCl, 0.05 % Nonidet P-40, 0.5 mM EDTA, 100 μg/ml BSA, 35 μg of poly(dI-dC)) at room temperature for 15 min, followed by six washes using 500 μl of NT2 buffer [28].

Pulled down DNA was then eluted by boiling and amplified again for a second cycle of systematic evolution of ligands by exponential enrichment (SELEX). For bait1, we performed 5 cycles of pulldown; for Bait-2, we performed 3 cycles. For sequencing, the product was amplified for 10 cycles using different combinations of indexing primers

Bait-1 input: N501-N701; Bait-1 output: N501-N702; Bait-2 input: N501-N704; Bait-2 output: N501-N705. Forward indexing primer, N501: AATGATACGGCGACCACCGAGATCTACACTAGATCGCTCGTCGGCAGCGTC. Reverse indexing primers: N701: CAAGCAGAAGACGGCATACGAGATTCGCCTTAGTCTCGTGGGCTCGG; N702: CAAGCAGAAGACGGCATACGAGATCTAGTACGGTCTCGTGGGCTCGG; N704: CAAGCAGAAGACGGCATACGAGATGCTCAGGAGTCTCGTGGGCTCGG; N705: CAAGCAGAAGACGGCATACGAGATAGGAGTCCGTCTCGTGGGCTCGG

Fifty 2.5 M base paired-end reads were then generated for each sample on an Illumina HiSeq Instrument.

### Analysis of SELEX-seq data

The Bait-1 SELEX-seq data was analyzed using the SELEX Bioconductor package [29]. A fifth-order Markov model was constructed using control Bait-1 sequences (no DUX4 pulldown) to predict the number of 16-mer sequences in each initial library as described [30, 31]. Sequence counts from the DUX4 pulldown of Bait-1 were compared expected counts as predicted using Markov model derived from control data to identify significantly enriched sequences. The top 20 enriched putative DUX4 binding sequences each contained one of the four 11 bp sequences described in Fig. 3.

## Results

### Measuring DUX4 transcriptional activation on identified target sequences

**Fig. 1:**
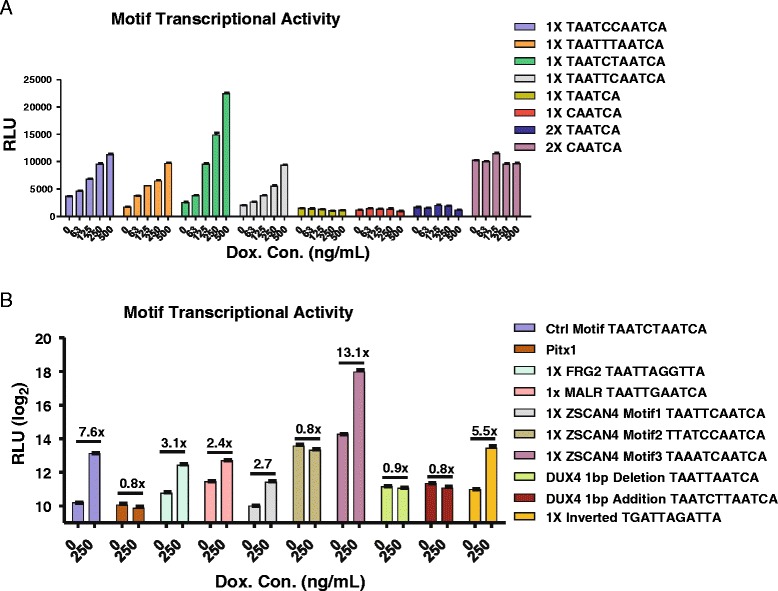
DUX4-dependent luciferase activity of the four flavors of the DUX4 ChIP-seq motif. **a** DUX4 dose dependence of the four flavors of the DUX4 ChIP-seq consensus (*left*) and “half-sites” in one or two copies (*right*). RLU, relative luciferase units (normalized to *Renilla* luciferase). **b** DUX4-induced expression of luciferase reporters bearing various previously described DUX4 recognition motifs and mutations. Fold change is shown above each pair of assays. Note the log scale

ChIP-seq analysis has identified a consensus containing two tandem TAAT motifs (TAAT[T/C][T/C]AATCA) [11]; however, the relative activity of DUX4 for the four individual motifs that match this consensus is unknown. We therefore began by transfecting luciferase reporters containing a single motif upstream of a minimal promoter on to 293T cells that we modified for doxycycline-inducible DUX4 expression. We also tested the activity of half-sites, having only the second TAAT motif with the terminal CA. This analysis showed that although all four variants could be recognized by DUX4, resulting in dox-dependent luciferase induction, the motif containing a central cytosine followed by a thymidine (TAATCTAATCA) had the greatest transcriptional activity in vivo (Fig. 1a). We therefore used this as our baseline positive control for other experiments. Half-sites did not show luciferase induction, although duplicating the CAATCA half-site apparently created a binding site for an unknown DUX4-unrelated activator (seen by dox-independent high level of background).

We next tested the activity of other putative DUX4-binding DNA sequences. The first sequence to which DUX4-binding was attributed was in the promoter of the murine *Pitx1* gene [10]. Two publications have shown band shift activity in nuclear extracts of cells transfected with DUX4 expression vectors [10, 27]. The similarity of the DUX4 homeodomains to those of Pax3 and Pax7 [22] which recognize TAAT sequences suggested that DUX4 might recognize a TAAT sequence within the *Pitx1* promoter. The sequence used in these studies does contain a TAAT, but it is quite different from the ChIP-seq motif, described above. It is actually two overlapping TAATs on different strands (i.e., TAATTA); therefore, we generated a luciferase reporter containing one copy of the full 30-bp sequence used in these studies. It has also recently been shown that *FRG2*, a gene upregulated in FSHD, has motifs recognized by DUX4 [32]; we included the motif of greatest match to the consensus motif in our comparison: TAACCTAATTA. We also included a sequence from the *MaLR* repetitive element that is a near match to the consensus (TAATTGAATCA, note the middle G which deviates from the consensus) because a number of DUX4 targets have ChIP-seq peaks in nearby *MaLR* elements [11, 33]. Because of recent mobility of *MaLR*, a subset of *MaLR* integrations are not shared between human and mouse, leading to concerns that a mouse model may not capture some relevant DUX4 target genes. One of the most strongly induced DUX4 target genes, *ZSCAN4*, has three potential DUX4 motifs. We included each of these motifs in our analysis. To complete this collection, we included two mutants in which the space between the two TAATs was increased or decreased by a single base, as well as the reverse complement of the control CT sequence to test the orientation dependence of the motif. The effect that background sequence may have on DUX4 binding is unknown, therefore, with the exception of the second and third *ZSCAN4* motifs, all of these sequences were embedded into the background sequence flanking the first *ZSCAN4* motif.

Cells were exposed to a relatively high dose of doxycycline (dox, 250 ng/ml) and transfected with each reporter (Fig. 1b). In the presence of dox, the control TAATCTAATCA sequence gave about 8-fold higher expression over background, and the reverse complement gave about 6-fold increased expression, demonstrating orientation independence of transcriptional activation by DUX4. Other active sequences included those from *FRG2* (~3-fold), *MaLR* (2.4×), and motifs 1 and 3 from ZSCAN4 (~3× and 13×, respectively) *ZSCAN4* motif 2, in which the A from the first TAAT was substituted, was inactive. The *Pitx1* sequence gave no activity. Neither did sequences in which the spacing between the two TAAT motifs was increased or decreased by a single base pair.

### Physical interaction between the DUX4 homeodomains and DNA

The lack of induction by the 30-bp *Pitx1* sequence was surprising, as it had previously been shown to interact with DUX4 in band shift assays. However, to date, published band shifts have been with nuclear extracts, not with purified protein. Therefore, given the striking lack of the consensus motif within the *Pitx1* sequence, we hypothesized that the previously documented band shifts might not be the result of a direct interaction with DUX4. We therefore expressed the DNA-binding N-terminal domain of DUX4, which contains both homeodomains, in *Escherichia coli* and tested the ability of the DUX4 DNA-binding domain to directly interact with the various target sequences studied above (studies on full-length DUX4 were precluded by the insolubility of the bacterially purified full-length protein). We synthesized a series of double-stranded oligos, all of which contained the putative DUX4 interaction motif within the context of the same arbitrary identical 14 bp of flanking sequence. Because the previously studied *Pitx1* sequence is 30 bp, we selected the central putative DUX4-interacting 13 nucleotides, containing the ATTAAT sequence, but we also tested the full 30-bp sequence independently. It is important to note that although the core ATTAAT sequence is conserved in the human *PITX1* gene, there are several sequence differences outside of this core.

**Fig. 2:**
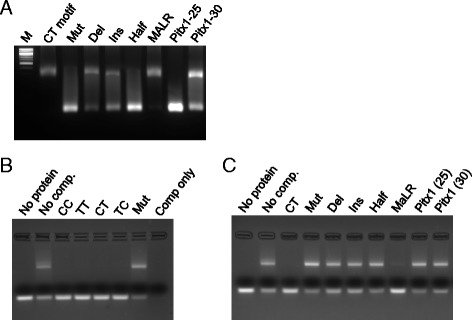
Evaluating protein-DNA interaction by electrophoretic mobility shift. **a** Direct band shift assays for various sequence motifs. The DNA is detected by EtBr staining. The CT probe contains the control TAATCTAATCA sequence and shows a complete shift to the slower-migrating form in the presence of an excess quantity of the DUX4 DNA-binding domain. Other oligos showed various shift efficiencies. **b** Competitive band shifts of the four flavors of the DUX4 ChIP-seq consensus motif. The CT probe is labelled with FAM; thus, the gel has no EtBr, and binding is competed with unlabeled oligos. All four flavors of the DUX4 ChIP-seq consensus effectively compete away binding to the FAM-CT probe when provided in excess, while the mutant sequence fails to compete. **c** Competitive band shifts of the sequences tested for direct band shifting (in **a**, above)

When this set of double-stranded oligos was simply mixed with excess protein, the band shift behavior of these sequences correlated roughly with their transcriptional activation potential: sequences that conferred DUX4-dependent luciferase induction also robustly shifted the probe, i.e., virtually all DNA was shifted, leaving no or almost no free probe (optimal “CT” motif and MALR, Fig. 2a). Note that the only molecules in this experiment are the protein and the DNA (visualized by ethidium bromide staining); therefore, the interaction is direct. The mutated DUX4 site, the half-site (i.e., TAATCA only) and the *Pitx1* core sequence in the context of the 25mer oligo all showed no shift. On the other hand, the motifs with a single base insertion or deletion as well as the full 30-bp *Pitx1* oligo showed some band shift activity, albeit with a significant unshifted band.

To more deeply investigate the relative affinity of DUX4 for these sequences, we performed competition experiments, in which we fluorescently labeled the “CT” consensus sequence (i.e., TAATCTAATCA) with FAM (carboxyfluorescein) and competed with the unlabeled sequences described above. For these experiments, the protein was limiting, thus even without competitor, only about 50 % of the FAM-labeled probe was shifted. When unlabeled and added in excess, all four flavors of the DUX4 ChIP-seq motif competed equally well (Fig. 2b). However, when the other unlabeled sequences described above were used in excess, outside of the CT control, only the *MaLR* sequence displayed competitive activity, and it was less competitive than the CT control (Fig. 2c). Thus, while the insertion and deletion mutants and the *Pitx1* 30-bp oligo do have some ability to interact with DUX4 when the protein is in excess, when the protein is not in excess, these sequences cannot effectively compete for interaction with DUX4.

### Determination of DUX4-binding preferences by SELEX

**Fig. 3:**
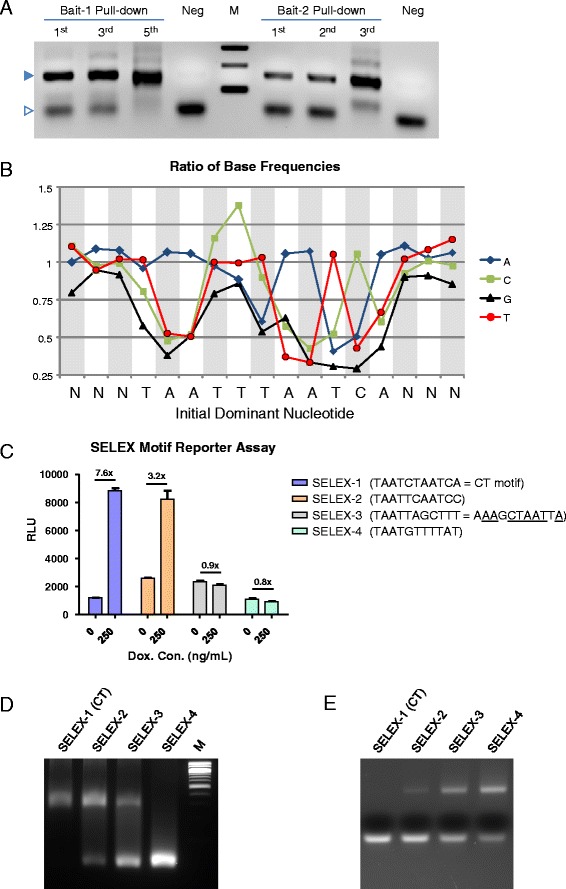
Discovery of DUX4-interacting motifs by SELEX. **a** PCR assay after indicated rounds of DNA pulldown. *Filled triangle* indicates the specific band; *open triangle* indicates primer dimers. Note that the amplified product is enriched over primer dimers by the last cycle shown. **b** Selection bias for each base at each position (base frequency in the pulled down sequence/base frequency in the control). **c** Luciferase reporter assays for the top four SELEX-identified sequences. **d** Direct band shift assays for the top 4 SELEX motifs. **e** Competitive activity of the top four SELEX motifs. Each unlabeled SELEX probe was used to compete the shift of a FAM-labeled probe bearing the CT motif

The experiments described above suggested that DUX4 interacts with variations on the double TAAT motif. To get a better sense of specific sequence preferences, we performed two SELEX-seq experiments. In the first experiment, we synthesized an oligo library based on the “TT” flavor of the ChIP-seq defined consensus: TAATTTAATCA, and at each base, we introduced an error rate of 9 % (i.e., 91 % of the correct nucleotide, 3 % of each incorrect nucleotide). We used the bacterially produced purified double homeodomain fragment to pull down this partially randomized oligo, and PCR amplified the pulled down products. In order not to completely lose lower-affinity sequences, we reiterated only to cycle 3 (Fig. 3a, Bait-2). A control, not pulled down sample, was also amplified three times to parallel the amplification to which the pulled down oligos were subjected. Following the third amplification, pulled down fragments were sequenced to a read depth of 2.5 M and the frequency of each base at each position was compared in the pulled down vs. control library. At every position except for position 5 (the nucleotide between the two TAATs), the ratio was >1, meaning that the consensus sequence was selected over the possible mutants (Fig. 3b). At position 5 however, a strong preference for C rather than T was observed. Thus, the CT consensus, the most transcriptionally active of the four flavors initially tested (Fig. 1a), was also the most preferred sequence in this partially randomized library. Position 4 showed a detectable, but weaker, preference for C. This preference was not strong enough to result in selection against the consensus T at position 4 (the ratio for T was still just above 1), but the C was clearly positively selected, and preferred over A or G. In addition to preferences within the 11-bp defined motif, we identified preferences for sequence flanking the motif. Most obviously, G was selected against at all three upstream and downstream positions, while there was a moderate preference for A and T at these positions.

In the second SELEX-seq experiment, our objective was to determine whether there were other sequences, not involving the double TAAT motif, with which DUX4 could strongly interact. Based on the preference of individual paired class homeodomains for a TAAT sequence [34], we randomized sequence around a core TAAT motif. We performed pulldowns of these oligos, PCR amplified, and reiterated four times (Fig. 3a, Bait-1). After the fifth pulldown, DNA was sequenced, as above. We analyzed these sequences using a SELEX analysis Bioconductor package (see the “Methods” section) and identified enriched motifs. The top selected motif turned out again to be the CT consensus (TAATCTAATCA). The next two were similar to the ChIP-seq consensus, and the fourth was a sequence that did not resemble a double TAAT. We tested each of these sequences in luciferase reporter (Fig. 3c) and direct and competitive band shift (Fig. 3d, e) assays. The top two SELEX motifs showed transcriptional activation activity as well as strong direct and competitive band shift activity, with the CT consensus not surprisingly giving the greatest activation and the best competition. The third motif, three mutations away from any ChIP-seq consensus, showed no transcriptional activity, weak band shift activity, and very weak competitive activity, while the fourth SELEX motif showed no activity in any assay. Thus, the second SELEX experiment did not find alternative motifs distinct from the double TAAT motif defined by ChIP-seq and furthermore independently identified the CT consensus as the most favorable of all randomly generated sequences.

### Synergy and spacing of clustered DUX4 binding sites

**Fig. 4:**
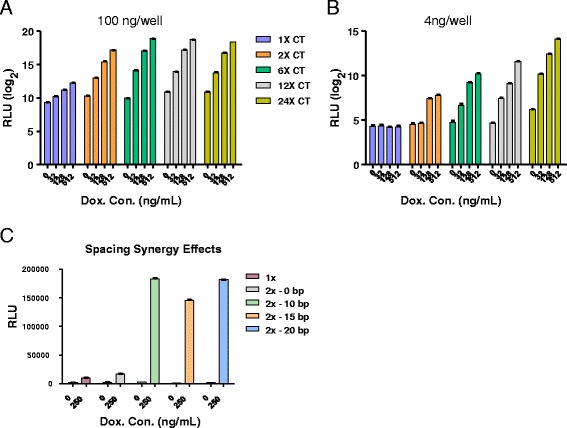
Evaluating reporters with multiple DUX4 binding sequences. **a** Dose-dependent activity of reporters with 1–24 copies of the CT motif at 100 ng of DNA/well. Note the logarithmic scale. **b** Dose-dependent activity of the same reporters at 4 ng/well. Synergy is demonstrated by the presence of activation with the 2× reporter but its absence with the 1× reporter at this concentration, as well as the continued increase in activity as copy number increases to 24×. **c** Transcriptional activity of reporters with two CT motifs spaced at different distances (indicated), compared to a reporter with a single CT motif

With multiple independent lines of evidence pointing to the CT flavor of the DUX4 motif being the most preferred DNA-binding sequence, we next multimerized the CT motif and evaluated whether targeting multiple copies of DUX4 upstream of a minimal promoter would have additive or synergistic effects on transcription. This was motivated by the observation that many upregulated DUX4 target genes, for example, *FRG2* and *ZSCAN4*, have multiple motifs to which DUX4 can bind [11, 32]. We generated reporters bearing 2, 6, 12, or 24 copies of the CT motif and evaluated dox-dependent luciferase activity in DUX4-inducible 293T cells. With transfection conditions optimized for detecting activity of the single copy reporter, additional copies of the DUX4-binding sequence clearly had a synergistic effect, seen most clearly going from one copy to two copies, where two copies were ~20-fold more potent than a single copy at the highest concentration of dox (Fig. 4a). Adding additional copies increased expression, but this effect plateaued at six copies. However, when much lower amounts of DNA were transfected, which gave no detectable expression of the single copy reporter, more copies clearly made for a much more sensitive reporter, and this effect did not plateau up to 24 copies (Fig. 4b), indicating that the previous peak at 6 copies was due to the amount of protein being limiting for the amount of DNA used.

The nature of the cooperativity seen with two sites could mean that two DUX4 molecules bind cooperatively, or it could simply be due to increased avidity for a DUX4-interacting transcriptional coactivator. To investigate the first possibility, we generated a series of constructs in which the spacing between the two sites was varied (Fig. 4c). The previous multimer experiments inserted 10 bp, approximately one full helical turn, between each 11-bp motif, meaning that neighboring DUX4 proteins are binding on the same side of the DNA molecule. First, we placed two CT motifs directly adjacent to one another. This construct behaved like a single site, suggesting that DUX4 occupies a footprint greater than the 11 bp that define its sequence specificity. We then generated a series of constructs with spacing increased in increments of half-turns: 15 bp, forcing the second DUX4 molecule to the opposite side of the DNA strand, and 20 bp, doubling the distance between the two DUX4 molecules. The transcriptional activation potential of all three constructs was roughly equivalent, suggesting that inter-molecular interactions of neighboring DUX4 molecules do not explain cooperativity.

## Discussion

These studies have revealed a number of interesting properties of the DUX4 protein. First, multiple lines of evidence point to the CT motif, TAATCTAATCA, as the most preferred and most transcriptionally active of sequences that match the DUX4 consensus, previously defined by interrogation of sequences under DUX4 ChIP-seq peaks [11]. Both a direct comparison of all four flavors of the consensus sequence and two different random screens based on the pulldown of DNA sequences by the DUX4 DNA-binding domain showed the CT motif to be the most active.

Second, one of the sequences historically used to measure DUX4 activity, a DNA sequence from upstream of the mouse *Pitx1* promoter, has little activity in the assays used here. It is possible that various optimizations could bring out some weak activity, and indeed, the full 30-bp oligo used in previous studies had some modest band shift activity in our hands (although no competitive activity), but in direct comparisons with various motifs based on the double TAAT sequence, the *Pitx1* sequence was inactive. Combined with the fact that this 30-bp sequence is not completely conserved between mouse and human, that the expected ChIP-seq peak was not found over the region corresponding to this 30-bp sequence in humans, or anywhere near *PITX1* [11], and the fact that *PITX1* was not found to be strongly upregulated by DUX4 in various human cell systems [11, 19] (or even mouse systems [22, 35, 36]), these data argue strongly against the model in which FSHD is caused by DUX4-mediated overexpression of *PITX1* [10].

Third, we find that single copies of DUX4 motifs are relatively ineffective at inducing transcription of target genes compared to double (or greater) copy numbers. This has implications for the relevance of mouse models for FSHD. It has been argued that the recent mobility of *MaLR* elements, which contain a single DUX4 motif, makes mouse a less than ideal model system to study DUX4 pathology, because the DUX4 binding sites created by a subset of these elements are in different places in the mouse genome compared to the human genome. Although the MaLR motif is a relatively strong individual motif, the fact that it is present only once within the *MaLR* repeat means that most *MaLR*-associated DUX4 binding sites do not have the capacity for high level DUX4-mediated target gene expression. Although individual DUX4 transcriptionally responsive sites have been identified within primate-specific *MaLR* set [33], there is nevertheless a large and significant overlap between the sets of mouse and human DUX4-responsive genes [37]. These considerations argue that the mouse is not particularly disadvantaged as a model organism in which to study FSHD.

The paired class of homeodomain proteins typically bind DNA as dimers, in which each homeodomain interacts with a TAAT sequence [34]. The dimer is symmetrical over the DNA axis; therefore, the second homeodomain of the dimer binds over the reverse compliment, i.e., ATTA. Thus, paired class homeodomains recognize the palindromic TAAT-N_*X*_-ATTA, and the orientation of the two homeodomains can be described as “head to head”. The gap between the TAAT and ATTA may be two nucleotides (a “P2” site), as in the case of Pax7 [26], or three nucleotides (a P3 site), as in the case of Pax6 [31]. It is tempting to speculate that because the DUX4 homeodomains are physically connected by only a short linker peptide, this might force the homeodomains to bind DNA in a head-to-tail fashion, meaning that the DUX4 motif could be considered an N1 site, i.e., a non-palindromic TAAT pair with a single-gap nucleotide. On the other hand, the 11-bp recognition sequence for DUX4 is the correct size for a P3 sequence, albeit one with a mismatch in position 10, rendering the ATTA into ATCA. Because the two homeodomains of DUX4 seem to have arisen from an internal duplication and are more similar to each other than to any other homeodomains, it seems improbable that they would recognize different core sequences. We await the structure of the DUX4-DNA complex to shed light on this question.

In the course of our study, we discovered certain sequences that have band shift activity when the protein is in excess, but failed to compete effectively when the protein is limiting and generally had no transcriptional activity. Because the DUX4 DNA-binding domain is actually two adjacent DNA-binding domains, we speculate that such band shifts may be due to DNA recognition by a single homeodomain only. Because half-sites did not have band shift activity, only certain TAAT sequences have this potential, most likely ones where the surrounding sequence tolerates and does not block positioning of the second homeodomain. This may open up an avenue for therapy development: if small molecules could be found that alter the interactions between the two homeodomains or between a homeodomain and DNA to stabilize such non-productive DNA binding, they might alter the DNA-binding specificity of DUX4 and thus diminish its toxicity.

## Conclusions

These studies demonstrate that the optimal DNA sequence preferred by DUX4 is the 11mer TAATCTAATCA (the CT motif). Other than a weak band shift seen when protein is in molar excess, DUX4 does not interact physically or functionally with the *Pitx1* promoter sequence, but it does interact with numerous variants of the optimal CT motif. Although transcriptional activation by DUX4 on targets with a single DUX4-binding motif is relatively weak, DUX4 shows tremendous synergy when a two or more sites are present in the same target. This implies that animal species such as mice, with partially divergent MaLR repeats (which carry single motifs only), are not more particularly disadvantaged for reasons over and above their conventional genetic divergence from humans, with regard to their suitability for modeling the physiological effects of DUX4 expression.

## Additional file

Additional file 1: Table S1. Oligos used in this study. (XLS 32.5 KB)

## Abbreviations

ChIP: chromatin immunoprecipitation
FAM: carboxyfluorescein
FSHD: facioscapulohumeral muscular dystrophy
SELEX: systematic evolution of ligands by exponential enrichment
